# Quality improvement interventions to prevent unplanned extubations in pediatric critical care: a systematic review

**DOI:** 10.1186/s13643-022-02119-8

**Published:** 2022-12-02

**Authors:** Krista Wollny, Sara Cui, Deborah McNeil, Karen Benzies, Simon J. Parsons, Tolulope Sajobi, Amy Metcalfe

**Affiliations:** 1 Department of Community Health Sciences, Cumming School of Medicine, University of Calgary, 2500 University Drive NW, PF 3239, Calgary, AB T2N 1N4 Canada; 2 Faculty of Nursing, University of Calgary, Calgary, AB Canada; 3 Alberta Children’s Hospital Research Institute, Calgary, AB Canada; 4 Alberta Children’s Hospital, PICU, Calgary, AB Canada; 5 Maternal Newborn Child and Youth Strategic Clinical Network, Alberta Health Services, Calgary, AB Canada; 6 Department of Pediatrics, Cumming School of Medicine, University of Calgary, Calgary, AB Canada; 7 Department of Obstetrics and Gynecology, Cumming School of Medicine, University of Calgary, Calgary, AB Canada; 8 Department of Medicine, Cumming School of Medicine, University of Calgary, Calgary, AB Canada

**Keywords:** Intensive care units, Pediatric, Patient safety, Systematic review, Extubation, Quality improvement, Critical care

## Abstract

**Background:**

An unplanned extubation is the uncontrolled and accidental removal of a breathing tube and is an important quality indicator in pediatric critical care. The objective of this review was to comprehensively synthesize literature published on quality improvement (QI) practices implemented to reduce the rate of unplanned extubations in critically ill children.

**Methods:**

We included original, primary research on quality improvement interventions to reduce the rate of unplanned extubations in pediatric critical care. A search was conducted in MEDLINE (Ovid), Embase, and CINAHL from inception through April 29, 2021. Two reviewers independently screened citations in duplicate using pre-determined eligibility criteria. Data from included studies were abstracted using a tool created by the authors, and QI interventions were categorized using the Behavior Change Wheel. Vote counting based on the direct of effect was used to describe the effectiveness of quality improvement interventions. Study quality was assessed using the Quality Improvement Minimum Quality Criteria Set (QI-MQCS). Results were presented as descriptive statistics and narrative syntheses.

**Results:**

Thirteen studies were included in the final review. Eleven described primary QI projects; two were sustainability studies that followed up on previously described QI interventions. Under half of the included studies were rated as high-quality. The median number of QI interventions described by each study was 5 [IQR 4–5], with a focus on guidelines, environmental restructuring, education, training, and communication. Ten studies reported decreased unplanned extubation rates after the QI intervention; of these, seven had statistically significant reductions. Both sustainability studies observed increased rates that were not statistically significant.

**Conclusions:**

This review provides a comprehensive synthesis of QI interventions to reduce unplanned extubation. With only half the studies achieving a high-quality rating, there is room for improvement when conducting and reporting research in this area. Findings from this review can be used to support clinical recommendations to prevent unplanned extubations, and support patient safety in pediatric critical care.

**Systematic review registration:**

This review was registered on PROSPERO (CRD42021252233) prior to data extraction.

**Supplementary Information:**

The online version contains supplementary material available at 10.1186/s13643-022-02119-8.

## Introduction

Endotracheal intubation is a life-saving intervention in the pediatric intensive care unit (PICU), used to assist children with breathing when they are unable to do so on their own. An unplanned extubation is defined as the uncontrolled and accidental removal of the endotracheal tube (ETT) and can adversely affect health outcomes [1–3]. Unplanned extubations are associated with increased number of ventilation days, longer length of stay in both the PICU and the hospital, increased medical expenses, and increased morbidity and mortality. Risk factors for unplanned extubation in the PICU include younger age [4–7], agitation [8–10], oral intubation [5], poor ETT fixation [11–13], copious secretions [4, 7, 14], patient procedures [15, 16], inadequate sedation [17, 18], repositioning/transport [10, 19], lack of restraints [4, 14, 20], and nurse-patient ratios [9, 14].

Recent studies have demonstrated rates of unplanned extubation in PICUs range between 0.11 and 6.4 events/100 airway days [1–3]. Quality improvement (QI) projects have been effective in decreasing the rate of unplanned extubations, usually targeting a rate less than 0.6/100 airway days [13, 21, 22]. *Quality improvement* has been defined as “systematic, data-guided activities designed to bring about immediate, positive changes in the delivery of health care in particular settings” [23]. The purpose of quality improvement is to deliver healthcare that is safe, effective, patient-centered, timely, efficient, and equitable [24]. Quality improvement involves continuous changes to practice at the local level, with the aim to improve patient and population health outcomes. Quality improvement interventions to prevent unplanned extubations have been primarily targeted towards staff education, standardizing sedation protocols, and standardizing procedures such as ETT securement, hygiene, and transport [10, 21, 22]. Due to the varying patient care practices and root-causes across individual PICUs, a variety of preventative practices have been implemented with inconsistent results.

The purpose of this article is to comprehensively review the literature that has been published on QI practices implemented to reduce the rate of unplanned extubations in critically ill children. Findings from this review will support clinical recommendations to prevent unplanned extubations, positively supporting patient safety in pediatric critical care.

## Methods

This systematic review was conducted and reported according to the Preferred Reporting Items for Systematic Review and Meta-analyses (PRISMA) 2020 reporting guidelines [25] (Supplemental Table 1) and was registered on PROSPERO (CRD42021252233) prior to data extraction.

### Eligibility criteria

We aimed to include literature on quality improvement interventions to reduce the rate of unplanned extubations in pediatric critical care. Studies were excluded if they 1) were not primary research (e.g., reviews, commentaries) 2); had a population focus outside of pediatric critical care (e.g., adult critical care or neonatal critical care) 3); did not include unplanned extubation rates; or 4) were only presented as an abstract.

### Information sources and search strategy

Literature searches were conducted in MEDLINE (Ovid), Embase, and CINAHL, from database inception to April 29, 2021. The search strategies for each database were developed using literature on the topic and in consultation with clinical experts. The search strategies were revised after reviewing preliminary search results and included synonyms for 1) pediatric intensive care, 2) quality improvement, and 3) unplanned extubations. No limitations on date or language were used for the search. The complete MEDLINE (Ovid) search strategy is shown in Supplemental Table 2. Citation searching of included articles was also used to hand-search for relevant literature. References were managed and de-duplicated in Covidence (Covidence systematic review software, Veritas Health Innovation, Melbourne, Australia).

### Selection process

After a subset of the team (KW, SC) achieved 100% agreement on a random sample of 10 citations, all titles and abstracts were reviewed independently and in duplicate by two reviewers (KW, SC). Any discrepancies were resolved by discussion. The full-text articles were then reviewed independently and in duplicate by two reviewers (KW, SC); again, any discrepancies were resolved by discussion, or the involvement of a third reviewer (AM) when necessary.

### Data collection process and quality assessment

Two reviewers (KW, SC) independently abstracted and reviewed data for each included study using a data abstraction form developed by the review team. Discrepancies were resolved through discussion with a third reviewer (AM). Information on study characteristics (e.g., year of publication, country, ICU type), QI intervention characteristics (e.g., description of interventions, time frame, barriers and facilitators), and outcomes (e.g., rates of unplanned extubation, statistical significance) were collected.

The Quality Improvement Minimum Quality Criteria Set (QI-MQCS) was used to assess the reporting of the quality improvement evaluation studies [26]. The QI-MQCS includes 16 domains, all of which were assessed at the study-level. Similar to other studies in this area, studies that report ≥ 14 domains are considered high quality [27].

### Data synthesis and analysis

Findings were analyzed and presented as a narrative synthesis. Vote counting based on the direct of effect was used to describe the effectiveness of quality improvement interventions [28]. All descriptive analyses were performed using the STATA 15 software (StataCorp. 2017. Stata Statistical Software: Release 15. College Station, TX: StataCorp LLC). Due to clinical heterogeneity between studies (patient and site characteristics), and the nature of QI interventions designed to target local root-causes, meta-analysis was not performed. Missing data were described as “not reported.”

Quality improvement interventions were categorized using the Behavior Change Wheel (BCW) [29]. The BCW is a model that was developed as a comprehensive synthesis of several other behavior change frameworks. It centers on the COM-B system, which recognizes that behavior change stems from capability, opportunity, and motivation [29]. Surrounding the COM-B system are intervention functions, which are then encompassed by policy categories. This acknowledges that policies (e.g., guidelines, regulations) can influence behavior through interventions (e.g., enablement, environmental restructuring) [29]. The described QI interventions within each study were coded as intervention functions (e.g., education, enablement, modeling) and/or policy categories (e.g., guidelines, regulations) [29]. A QI intervention as described by authors could fall into more than one category. Coding was done independently and in duplicate; any discrepancies were resolved through discussion.

## Results

### Study selection

**Fig. 1:**
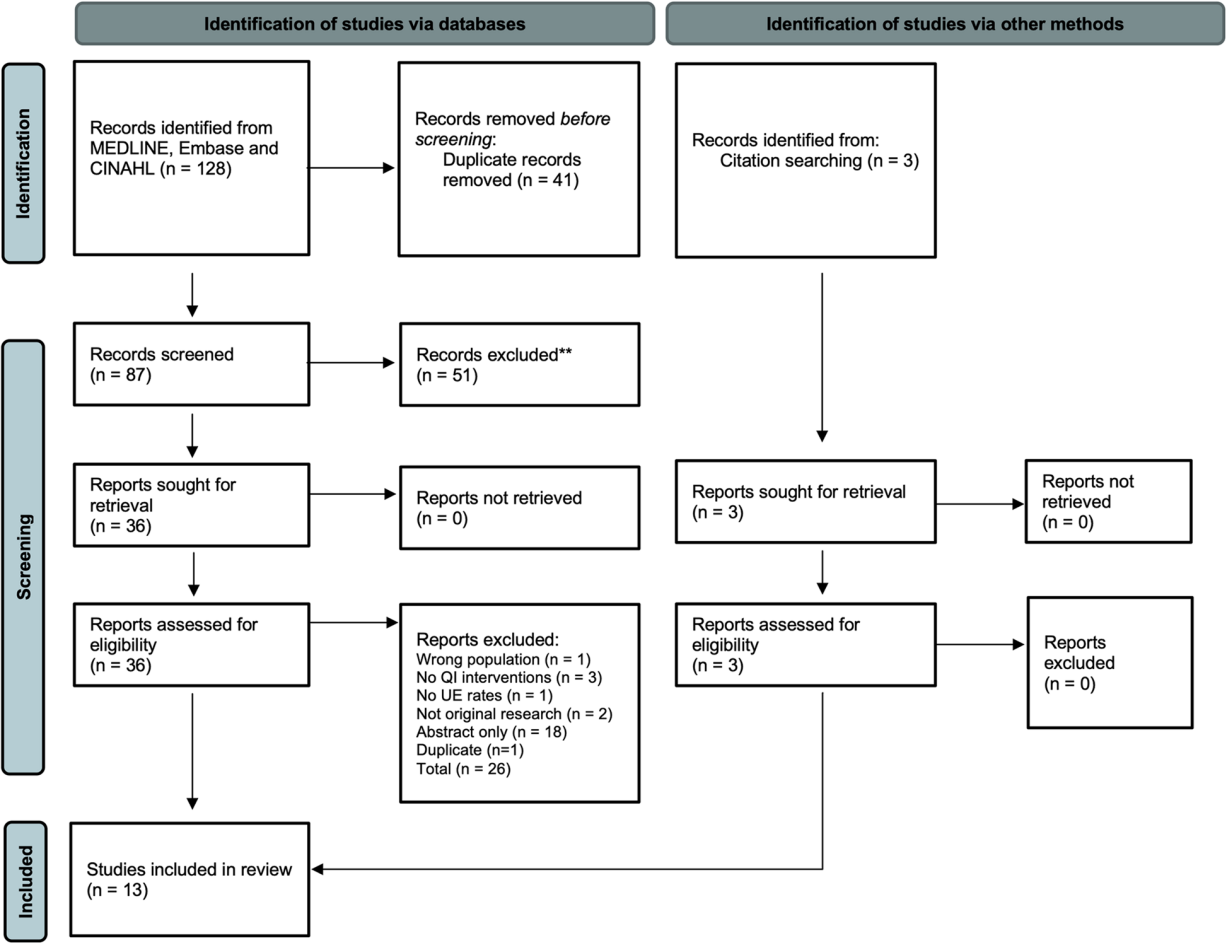
PRISMA 2020 flow diagram

The search strategy identified 128 records. After duplicates were removed, we screened 87 unique abstracts and reviewed 36 full-text articles; 26 full-text articles were excluded, the most common reasons being abstract-only publications or not describing quality improvement interventions. See PRISMA flow diagram (Fig. 1). Citation searching identified 3 potential studies, all of which were included. The frequency of disagreements and agreements between reviewers for the full-text screen was calculated at 0.880 using Cohen’s Kappa statistic.

### Study characteristics

Thirteen studies were included in the review. Study characteristics are summarized in Table 1 and Supplemental Table 3. The studies were published between 2004 and 2020 and took place in the USA (*n* = 9; 69.2%), Canada (*n* = 2; 15.4%), Brazil (*n* = 1; 7.7%), and Argentina (*n* = 1; 7.7%). Most studies were conducted in a PICU (*n* = 9; 69.2%); three studies took place in both a PICU and a cardiac intensive care unit (CICU), and one took place in a CICU only. Reported unit sizes ranged from 5 to 26 beds per unit. Twelve of the 13 studies took place at a single center; one was a multi-center study that included PICUs and CICUs from 43 children’s hospitals [21]. The majority were prospective cohort studies (*n* = 10; 76.9%); two studies had retrospective cohort designs, and one was mixed-methods.

**Table 1:** Study characteristics

Author	Year	Country	Unit Type(s)	Single vs. Multi-Centre	Unit Size (Bed N=)	Study Design	SQUIRE guidelines	Sustainability Study
Dechert et al.	2004	United States	PICU	Single	16	Prospective		
Popernack et al. [18]	2004	United States	PICU	Single	NR	Prospective		
da Silva et al. [6]	2008	Brazil	PICU	Single	5	Prospective		
Rachman et al. [30]	2009	USA	PICU	Single	10	Prospective		
Kaufman et al. [31]	2012	USA	PICU CICU	Single	PICU=26 CICU=16	Prospective		
Meregalli et al. [11]	2013	Argentina	PICU	Single	11	Prospective		
Rachman et al. [17]	2013	USA	PICU	Single	10	Prospective		
Menon et al. [12]	2015	Canada	PICU	Single	NR	MM		
Tripathi et al. [10]	2015	USA	PICU	Single	20	Prospective		
Al-Abdwani et al. [1]	2018	Canada	PICU CICU	Single	NR	Retrospective		
Kandil et al. [22]	2018	USA	PICU	Single	19	Prospective		
Censoplano et al. [13]	2020	USA	CICU	Single	16	Retrospective		
Klugman et al. [21]	2020	USA	PICU CICU	Multi	NR	Prospective		

*PICU* Pediatric ICU, *CICU* Cardiac ICU, *NR* Not reported, *MM* Mixed methods

### Quality improvement initiatives

Two studies [13, 17] were designed to assess the sustainability of quality improvement initiatives, and were analyzed separately from the other 10 primary studies. Both assessed the sustainability of QI studies included in this review [30, 31].

Studies were conducted over a period of 11–110 months, which, for most, included pre- and post-implementation data collection. Of the studies that explicitly reported the implementation period, interventions were implemented over a range of 3–24 months. Rates of unplanned extubation were collected up to 6–48 months post-intervention. QI interventions are detailed in Supplemental Table 3.

The median number of interventions implemented in each study was 5 [IQR: 4–5] (Table 2). Most intervention functions implemented were categorized as environmental restructuring (e.g., standardizing practice through a guideline, protocol, or algorithm; *n* = 10) or education (e.g., workshops, education days; *n* = 8). Most of the policy categories were classified as guidelines (e.g., sedation algorithms or tube securement protocols; *n* = 10) and communication/ marketing (e.g., posters, e-mails; *n* = 8). Guidelines were mainly focused on sedation practices (*n* = 5), standardization of care for intubated patients (*n* = 5), and ETT fixation/ securement (*n* = 5); many sites implemented more than one guideline as part of the QI project (*n* = 7).

**Table 2:** QI Interventions categorized using the behaviour change wheel

	Intervention Functions						Policy Categories					
Author (Year)	Education	Persuasion	Training	Enablement	Modeling	Environmental Restructuring	Guidelines (and description)	Environmental/ Social Planning	Communication/ Marketing	Regulation	Total (N=)	Change to UE Rate
Dechert et al. (2004)							Vent weaning Sedation Protocol				4	⇓ *
Popernack et al. (2004) [18]							Sedation Algorithm				2	⇓ *
da Silva et al. (2008) [6]							Standardization (care) Sedation Protocol				4	⇓ *
Rachman et al. (2009) [30]							Tube fixation policy				4	⇓ *
Kaufman et al. (2012) [31]							Standardized handover Sedation Protocol				5	⇓ (PICU) ⇓ * (CICU)
Meregalli et al. (2013) [11]							Tube fixation policy				5	⇓ *
Menon et al. (2015) [12]							Standardization (care) Tube fixation policy				6	=
Tripathi et al. (2015) [10]							Standardization (care) Sedation				5	⇓
Al-Abdwani et al. (2018) [1]											6	⇓
Kandil et al. (2018) [22]							Tube fixation policy Protocol for high-risk situations				4	⇓
Klugman et al. (2020) [21]							Tube fixation policy Protocol for high-risk situations				5	⇓ * (PICU) = (CICU)

⇓ Decrease; = No change; * Statistically Significant at p<0.05; PICU: Pediatric Intensive Care Unit; CICU: Cardiac Intensive Care Unit

No intervention was categorized as incentivization, coercion, restrictions, legislation, service provision or fiscal measures, so these categories were left out of this table

Reported barriers to implementation included decreased baseline uniformity of practice, nurses being pulled away from the bedside, and frequent rotation of staff (Supplemental Table 3). Facilitators included increased nursing autonomy, culture change focused on safety, improved multi-disciplinary communication, and team leadership.

### Unplanned extubation rates

All studies reported pre- and post-QI intervention unplanned extubation rates. Pre-intervention rates ranged from 0.44 to 6.40 events/100 airway days. Post-intervention rates ranged from 0 to 2.59 events/100 airway days. Ten studies reported a decreased rate of unplanned extubations after the QI intervention (Table 2). Of these, seven studies reported a statistically significant (*p* < 0.05) decrease; three studies did not describe statistical significance. Two studies observed statistically significant decreases in one unit, but not in the other (e.g., PICU vs CICU) [21, 31].

### Sustainability studies

Two studies reported the sustainability of QI interventions to prevent unplanned extubations. Rachman at al [17]. reported the rates of unplanned extubation 9 years after they implemented QI interventions [30]. Similarly, Censoplano et al. [13] studied the rates of unplanned extubations 7 years after QI interventions were implemented [31]. Both sustainability studies observed an increase in the rate of unplanned extubations (0 to 0.4 and 1 to 1.5/100 airway days, respectively); however, these increases were not statistically significant.

### Quality assessment

The median quality score using the QI-MQCS was 13.4 (IQR: 12–16). Five of the eleven primary studies were rated high quality (Supplemental Table 4). Many of the domains were reported by all studies, including organizational motivation, intervention rationale, intervention description, study design, comparator, data source, timing, health outcomes, and limitations. The domains that were reported the least include adherence/fidelity, sustainability, and spread.

## Discussion

This systematic review and narrative synthesis of quality improvement initiatives to prevent unplanned extubations in pediatric critical care identified interventions that have been used to change behavior, including education, environmental restructuring, guidelines, and communication/marketing. Most studies observed a decrease in unplanned extubation rates after the implementation of QI interventions, and two sustainability studies found that the decreased rate remained stable years later.

Most studies described several QI interventions implemented together to change healthcare provider behavior. Often, adverse events and patient safety issues, such as unplanned extubations, have multifactorial causes [1, 21, 32]. Previous work in the area of pediatric patient safety has outlined an approach to patient safety which includes 1) identifying the epidemiology of events/errors, 2) integrating a culture of patient safety, and 3) creating and implementing patient safety solutions [33]. This multipronged implementation approach was observed in the studies included in this review. Guidelines or protocols often change behavior through environmental restructuring, which was observed as a frequently used behavior change technique. Marketing and communication, alongside education and training, were also frequently implemented, targeting patient safety solutions to change. A similar review of QI interventions to reduce the rate of unplanned extubations in adult critical care also found most QI programs involved multiple interventions, including standardization of procedures (guidelines), and education [2]. The guidelines and procedures were also targeting similar foci, including sedation, nurse-to-patient ratios, and endotracheal tube fixation [2]. The policy categories and intervention functions that were not identified in this review include incentivization, coercion, restrictions, legislation, service provision, or fiscal measures. A similar review in critical care quality improvement also identified these as interventions that are less frequently used to enact change in this setting [34].

The included multi-site study by Klugman et al. [21] described a nationwide quality improvement initiative that included 43 sites across the USA, led by the Children’s Hospitals’ Solutions for Patient Safety (SPS)—a network dedicated to eliminate serious harm in pediatric care [35, 36]. An “Unplanned Extubation Quality Improvement Bundle” was developed, and sites were encouraged to implement the bundle locally using the Model for Improvement or Lean Six Sigma [21]. The bundle—a set of evidence-based practice suggestions—standardized care but allowed for site-specific contextual factors and preferences. Overall, the hospitals with higher bundle compliance had greater reduction in the rates of unplanned extubations. Bundles to reduce the rate of unplanned extubations have been effective in other patient populations, such as in neonatal intensive care [37].

Less than half of the studies were rated as high-quality using the QI-MQCS [26]. The domains that were reported the least were adherence/fidelity, sustainability, and spread. Adherence/fidelity refers to the process measures within a QI study, such as how many learners attended an education session, or how often healthcare providers followed the guideline [26]; process measures allow for interpretation about how well an intervention was implemented as planned [38]. This lowers the quality of a QI study by omitting important contextual details that prevent comparisons between studies and reproducibility. Sustainability refers to the potential for intervention maintenance [26]. A discussion about sustainability is important for understanding whether the outcome will persist, as long-term sustainability is the goal. Finally, spread refers to the ability of the intervention to be replicated in other settings [26]. This concept is essential for clinicians to be able to conceptualize how the intervention could be implemented at their own site, considering contextual factors are key to an intervention’s success. Overlooking the concept of spread may limit future generalizability.

The Standards for Quality Improvement Reporting Excellence (SQUIRE) guidelines were first published in 2008 and then revised in 2016 [39]. Although most studies were published after the SQUIRE guidelines were released, none of the primary QI studies referenced the guidelines, and only one sustainability study did [17]. The use of the SQUIRE guidelines in conducting and reporting future research will increase study quality and transparency.

This systematic review is not without limitations. Due to the heterogeneous nature of QI interventions and their focus on local causes, facilitators, and barriers to change, we were unable to meta-analyze the results. To allow readers to interpret each QI study within the context of the local environment, we presented background information such as unit size and type, location, QI interventions, timeframes, and interpreted barriers and facilitators to change (Supplemental Table 3). We caution readers to thoughtfully consider how these results may or may not align with their own local context, and use the information to help inform (but not dictate) future implementation strategies.

This review, as conducted, reflects the original protocol registered in PROSPERO (May 2021). Of note, this manuscript provides more detailed inclusion and exclusion criteria than the original protocol described. Originally, the authors considered including only English language publications; however, after recognizing there was only one non-English publication that was eligible (Meregalli et al. [11]), the authors opted to remove non-English language as part of the exclusion criteria. Mirroring the protocol, only three databases were searched, which may have impacted the ability to find additional publications on the topic. However, we feel as though most journals and publications on pediatric critical care adverse events and quality improvement would be indexed in the three databases that were searched and that any additional studies would have been caught using citation searching of included articles.

We did not include preliminary findings from conference proceedings, as not enough contextual information would be presented for analysis in an abstract. This may have limited the information located on the topic. Furthermore, there is risk of publication bias: the increased likelihood of publication with significant results [40]. We found and included one study that did not demonstrate decreased rates of unplanned extubations after implementing QI interventions [12]; however, all other studies demonstrated the intended decrease in unplanned extubation rates, suggesting potential publication bias. Including more studies with null findings would allow for a comparison between interventions that did and did decrease the rates of unplanned extubations in pediatric critical care.

This systematic review has several strengths. Using the QI-MQCS, we were able to comprehensively assess the quality of the included studies using a validated evaluation tool specific to QI research [26]. This also highlights where future literature on the topic could improve. Additionally, this is the first study to systematically synthesize the literature available on QI interventions to reduce the rate of unplanned extubations in pediatric critical care. This systematic review adds to the literature by providing a comprehensive overview of the QI interventions to decrease rates of unplanned extubations in pediatric critical care. Patient safety is an ongoing strategic priority at most children’s hospitals, with unplanned extubations listed as a key quality indicator [35, 41, 42]. Future research in this area should continue to explore the sustainability of QI interventions to decrease the rate of unplanned extubations in pediatric critical care, to be able to observe if improved rates are maintained over time.

## Conclusions

This systematic review narratively describes QI interventions to decrease the rate of unplanned extubations in pediatric critical care. Most studies described several interventions used together to decrease the rate of unplanned extubation, with the most frequent being guidelines, environmental restructuring, education, training, and communication. The findings from this review must be interpreted in the context of the study’s limitations, including the possibility of missing literature. Despite this, the description of the included studies’ interventions used to decrease unplanned extubation rates can be used by other hospitals aiming to do the same; pediatric critical care units can use this information to design and implement local QI interventions based on the contextual factors at their own sites.

## Supplementary Information

**Additional file 1: Supplemental Table 1.** PRISMA 2020 Checklist.

**Additional file 2: Supplemental Table 2.** MEDLINE (Ovid) Search Strategy.

**Additional file 3: Supplemental Table 3.** Study Characteristics and Quality Improvement Interventions.

**Additional file 4: Supplemental Table 4.** Study Quality Assessment Using QI-MQCS.

## Data Availability

Not applicable. No additional data available. Statistical code will be shared upon reasonable request to the corresponding author.

## Abbreviations

BCW: Behavior Change Wheel
CICU: Cardiac intensive care unit
ETT: Endotracheal tube
PICU: Pediatric intensive care unit
PRISMA: Preferred Reporting Items for Systematic Review and Meta-analyses
QI: Quality improvement
QI-MQCS: Quality Improvement Minimum Quality Criteria Set
SPS: Solutions for Patient Safety
SQUIRE: Standards for Quality Improvement Reporting Excellence
