# Supplementary material for: Quality improvement interventions to prevent unplanned extubations in pediatric critical care: a systematic review
Source: Syst Rev. 2022 Dec 2;11:259. doi: 10.1186/s13643-022-02119-8 (PMC9717500; doi:10.1186/s13643-022-02119-8)
Supplement: Supplementary file 2 — Additional file 2: Supplemental Table 2. MEDLINE (Ovid) Search Strategy. [file 13643_2022_2119_MOESM2_ESM.docx]

Supplemental Table 2: MEDLINE (Ovid) Search Strategy

|  | **Search Terms** |
| --- | --- |
| **Population:**  Pediatric Intensive Care | exp child/ or exp infant/ or adolescent/ or exp pediatrics/ or (pediatric* or paediatric* or child* or infan* or baby or babies or toddler* or boy* or boys or girl* or teen* or youth* or adolesc*).mp. or (child* or adolesc* or pediat* or paediat*).jn. or exp "congenital, hereditary, and neonatal diseases and abnormalities"/ or exp infant/ or (newborn* or congenital* or infan* or baby or babies).mp.  AND  exp Intensive Care Units/ or exp Critical Care/ or exp Critical Illness/ or PICU*.mp or critical* ill*.mp or intensive care.mp or critical care.mp Or (p?ediatric icu or p?ediatric icus).tw,kf |
| **Intervention:**  Quality Improvement | exp Quality Improvement/ or quality improv*.mp or LEAN.mp or six-sigma.mp or Total Quality Management/ or quality manag* or pdsa.mp |
| **Outcome:**  Unplanned Extubation | (unplanned extubation or accidental extubation or unint*extubation or self extubation or unexpected extubation or inadvertent extubation or spontaneous extubation).mp. |
