# Supplementary material for: Quality improvement interventions to prevent unplanned extubations in pediatric critical care: a systematic review
Source: Syst Rev. 2022 Dec 2;11:259. doi: 10.1186/s13643-022-02119-8 (PMC9717500; doi:10.1186/s13643-022-02119-8)
Supplement: Supplementary file 3 — Additional file 3: Supplemental Table 3. Study Characteristics and Quality Improvement Interventions. [file 13643_2022_2119_MOESM3_ESM.docx]

Supplemental Table 3: Study Characteristics and Quality Improvement Interventions

| Author (Year) | Unit Type | Single vs. Multi-Centre | Unit Size (Bed N=) | Number of QI Interventions Implemented* | QI Interventions | Intervention timing (months) | QI Frame-works | Process Measures | Pre-QI  UE Rate (/100 intubation days) | Post-QI  UE Rate (/100 intubation days) | Increase, Decrease or No change | Statistically Significant  (p<0.05) | Barriers | Facilitators | Study Quality Assessment (QI-MQCS)  /16 |
| --- | --- | --- | --- | --- | --- | --- | --- | --- | --- | --- | --- | --- | --- | --- | --- |
| Dechert et al. (2004) | PICU | Single | 16 | 4 | Phase 1: Data collection, root-cause analysis; Phase 2: Education, protocol-directed weaning;  Phase 3: Standardized sedation protocol | Phase 1: 24m; Phase 2: 12m; Phase 3: 12m  Total: 48m | PDSA | NR | 1.5 | 0.8 | Decrease | Yes | NR | Staff education, staff-to-patient workloads, management protocols | 12 |
| Popernack et al. (2004) | PICU | Single | NR | 2 | Data collection,  Standardized sedation algorithm | 60m observational period;  12m implementation period;  48m evaluation period  Total:  110m | NR | NR | 0.44-0.63 | 0.00-0.19 | Decrease | Yes | NR | Management protocols/guidelines, improved nursing autonomy, multidisciplinary communication | 13 |
| da Silva et al. (2008) | PICU | Single | 5 | 4 | Data collection, root-cause analysis, education, standardizing procedures (tracheal tube fixation, tube suctioning, hygiene, and transport), identifying patients at high risk of UE, and standardized sedation protocol. | Phase 1: 1m;  Phase 2: 24m;  Phase 3: 1m;  Phase 4: 12m  Total:  38m | PDSA | Primary investigator regularly visited unit to ensure compliance | 2.9 | 0.6 | Decrease | Yes | NR | NR | 14 |
| Rachman et al. (2009) | PICU | Single | 10 | 4 | Period 1:  Data collected, determined rate and causes of UE  Period 2:  Determined rate/cause of UE, education,  Standardized endotracheal tube taping policy, data collection | Intervention 7m;  6m evaluation period  Total:  13m | PDSA | Practitioners had to demonstrate compliance to taping protocol after staff education | 6.4 | 1.0 | Decrease | Yes | NR | NR | 13 |
| Kaufman et al. (2012) | PICU  CICU | Single | PICU=26  CICU=16 | 5 | Standardization of endotracheal tube taping practices upon admission, improve patient handoffs, root-cause analysis of unplanned events, re-examination of sedation practices, transparency of performance measures | Preintervention stage: 9m;  Intervention stage: 8m;  Post-intervention:  7m  Total: 24m | PDSA | Auditing of unit-specific compliance | PICU: 0.8 CICU: 0.74 | PICU: 0.29 CICU: 0.0 | Decreased | PICU-no  CICU-yes | Difficult to determine rates of compliance in each unit-may have contributed to difference in rates of events, less uniformity of practice in the PICU | Accurate data collection and tracking, protocols, staff education | 16 |
| Meregalli et al. (2013) | PICU | Single | 11 | 5 | Training of PICU staff (explain purpose of study, raise awareness of consequences of UE); Standardization of endotracheal tube securement and assessment. | Preintervention:5m;  Intervention: 6m  Total: 11m | NR | NR | 2.3 | 0.9 | Decreased | Yes | NR | NR | 11 |
| Rachman et al. (2013) | PICU | Single | 10 | NA | N/A (Sustainability Study)  Original study: Rachman et al. (2009) | Follow-up study: 9 years after QI interventions | NA | NA | 1.0 | 1.5 | No change | No | NR | NR | NA |
| Menon et al. (2015) | PICU | Single | NR | 6 | Phase 1: Data collection  Phase 2: develop data tracking tools, staff education, standardized monitoring routines, standardization of tracheal tube fixation, tube suctioning, and patient transport | Phase 1: 24m;  Phase 2: 3m;  Phase 3: 9m  Total: 36m | NR | Compliance verified by checking with resident each morning | 0.9 | 0.9 | No change | No | Nurses being pulled away from bedside | Staff education/training, used best practice guidelines from literature and consultation with PICU Partnership Council | 16 |
| Tripathi et al. (2015) | PICU | Single | 20 | 5 | Data collection, interim analysis, staff education, care policy created (includes standardization of sedation assessment and documentation policy, tube suctioning) | Period 1: 6m;  Data collection continued for 6m during 6m implementation.  Total: 12m | NR | NR | 3.55 | 2.59 | Decreased | NR | NR | NR | 12 |
| Al-Abdwani et al. (2018) | PICU  CICU | Single | NR | 6 | Full-time Quality Lead, data collection, extubation huddle, education, change in securement device | 60m | NR | NR | 0.98 | 0.37 | Decreased | NR | NR | Ongoing data collection/improvement strategy using feedback from stakeholders | 10 |
| Kandil et al. (2018) | PICU | Single | 19 | 4 | Phase 1: data collection  Phase 2: ET securement standardization, safety culture, bedside reminders, multidisciplinary review of all events, high-risk situation strategies (protocol for bedside tube manipulation and protocol for patient transports) | Phase 1: 7m;  Phase 2: 13m;  Phase 3: 21m  Total: 41m | PDSA | Compliance with process bundles collected from 2016-2018, averaged 93.7% | 0.9 | 0 | Decreased | NR | NR | Implemented improvement efforts across entire Children’s hospital setting;  Staff leadership to change work culture (culture of safety).  Joined collaborative with support from hospital leadership that provided national network of support. | 16 |
| Censoplano et al. (2020)* | CICU | Single | 16 | NA | NA (Sustainability Study)  Original study: Kaufman et al. (2012) | Follow-up study: 7 years after QI interventions | NA | NA | 0 | 0.4 | Increased | NR | Healthcare members rotate frequently (inconsistency of staff) | Staff training/ education | NA |
| Klugman et al. (2020) | PICU  CICU | Multi | NR | 5 | Standardized anatomic reference points and securement methods, protocol for high-risk situations, multidisciplinary apparent cause analyses | Preliminary period: 8m;  Implementation period: 11m;  Follow-up: 15m  Total: 34m | Model for Improvement, Lean Six Sigma | Compliance with bundle – submitted monthly | PICU: 0.729  CICU:  0.281 | PICU: 0.579  CICU: 0.281 | PICU: Decreased  CICU: No change | Yes | NR | Standardized bundle increased consistency of care.  Collaborative provided structure/resources to guide QI, accelerating and sustaining improvement  Team leadership and support staff.  Staff education | 14 |

Acronyms: CICU – cardiac intensive care unit; m – months; NR – not reported; NA – not applicable; PICU – pediatric intensive care unit; PDSA – Plan-do-study-act; QI – quality improvement; QI-MQCS - Quality Improvement Minimum Quality Criteria Set

*Classified using the Behaviour Change Wheel
