# Supplementary material for: Quality improvement interventions to prevent unplanned extubations in pediatric critical care: a systematic review
Source: Syst Rev. 2022 Dec 2;11:259. doi: 10.1186/s13643-022-02119-8 (PMC9717500; doi:10.1186/s13643-022-02119-8)
Supplement: Supplementary file 4 — Additional file 4: Supplemental Table 4. Study Quality Assessment Using QI-MQCS. [file 13643_2022_2119_MOESM4_ESM.docx]

Supplemental Table 4: Study Quality Assessment Using QI-MQCS

| Author | Organisational Motivation | Intervention Rationale | Intervention Description | Organizational Characteristics | Implementation | Study Design | Comparator | Data Source | Timing | Adherence/ Fidelity | Health Outcomes | Org. Readiness | Penetration/ Reach | Sustainability | Spread | Limitations | Domains (Total) |
| --- | --- | --- | --- | --- | --- | --- | --- | --- | --- | --- | --- | --- | --- | --- | --- | --- | --- |
| Dechert et al. (2004) | 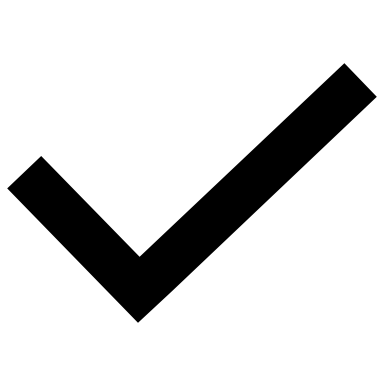 | 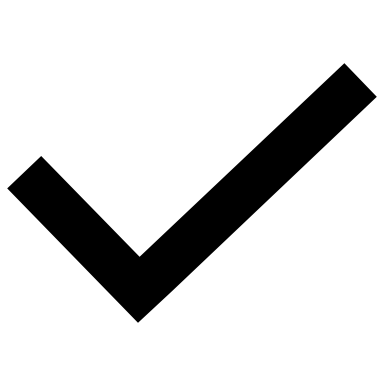 | 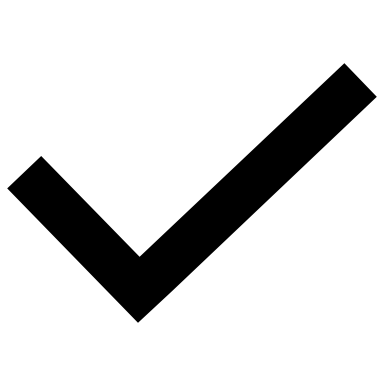 | 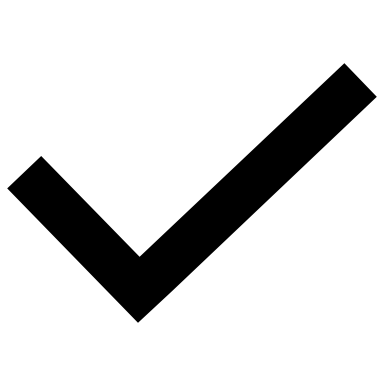 | 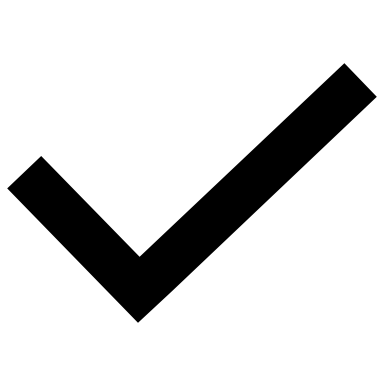 | 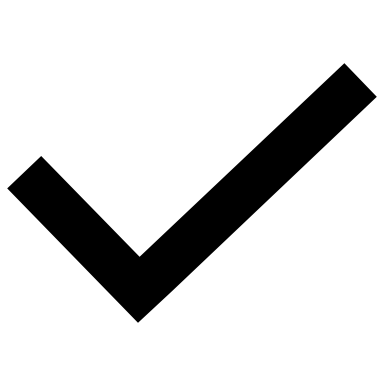 | 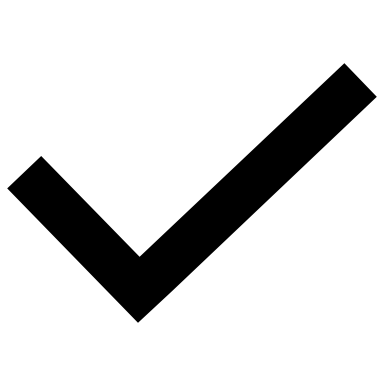 | 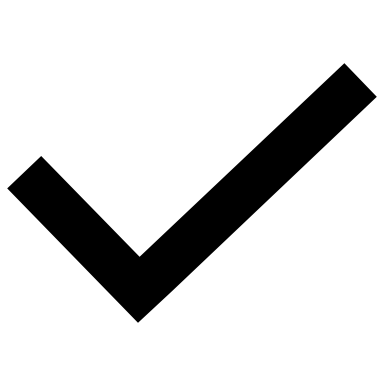 | 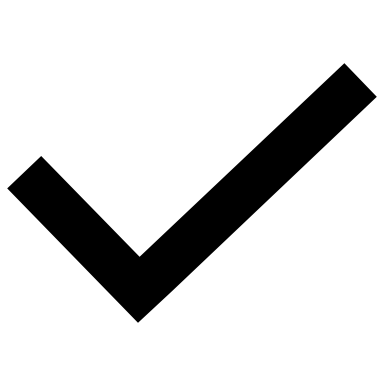 |  | 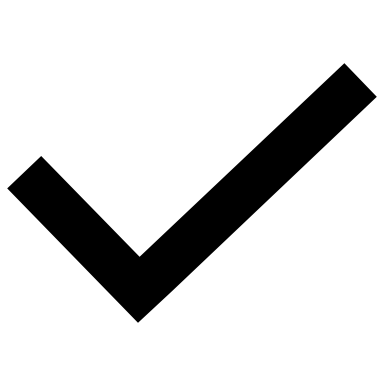 | 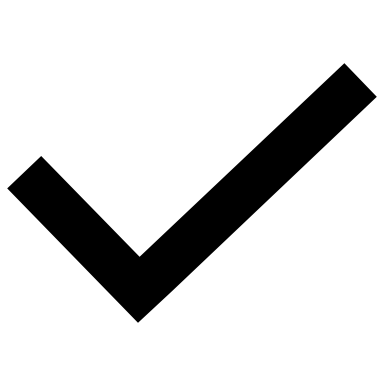 | 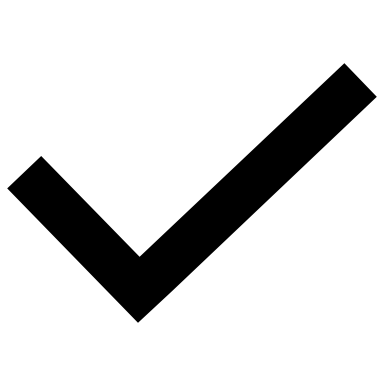 |  |  | 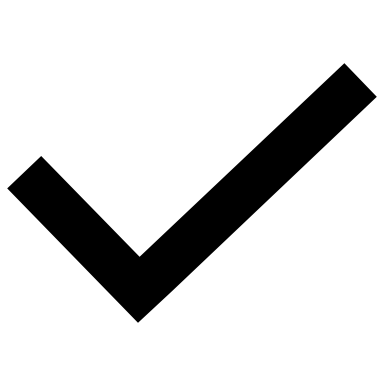 | 12 |
| Popernack et al. (2004) | 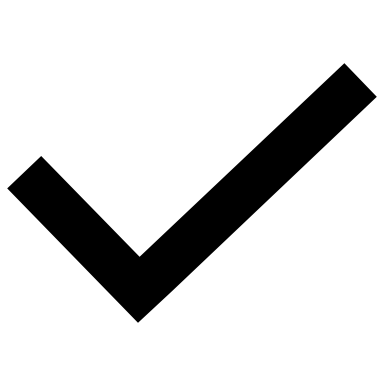 | 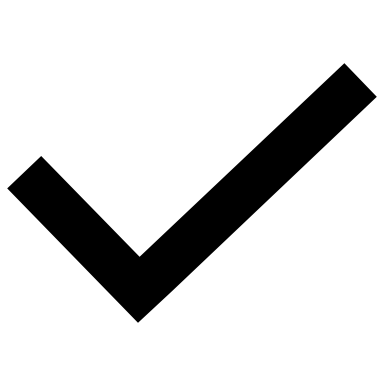 | 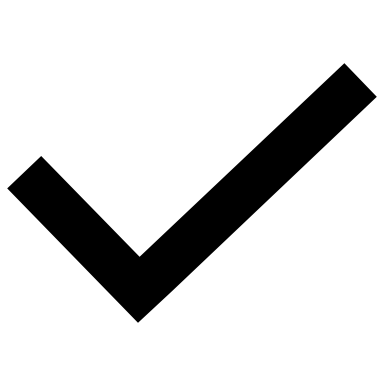 | 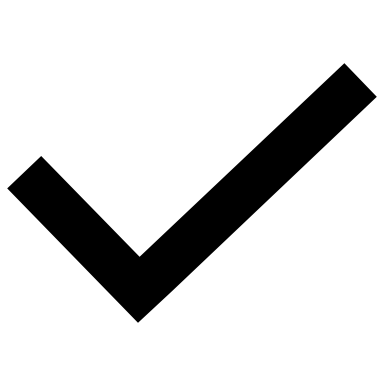 | 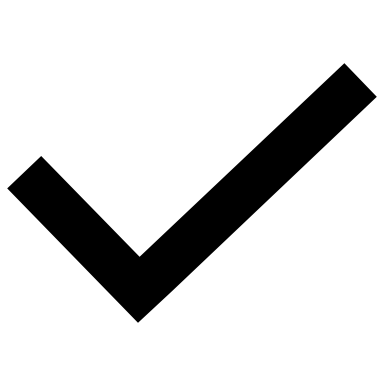 | 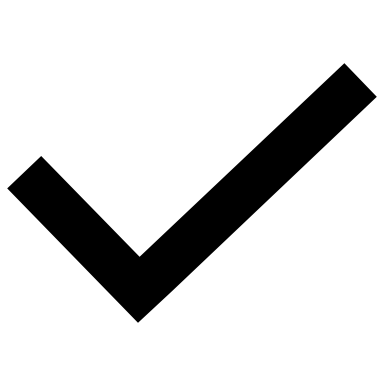 | 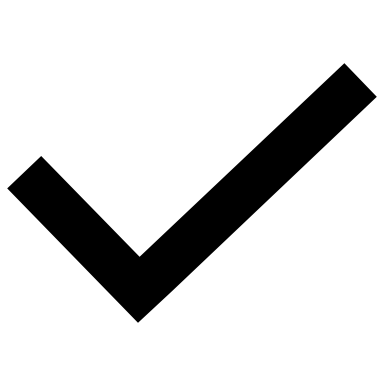 | 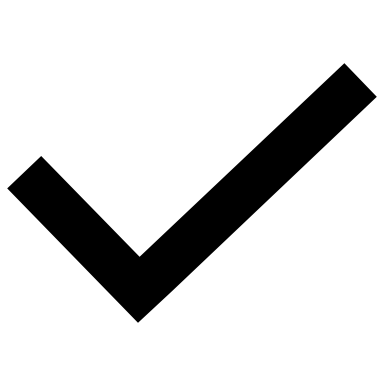 | 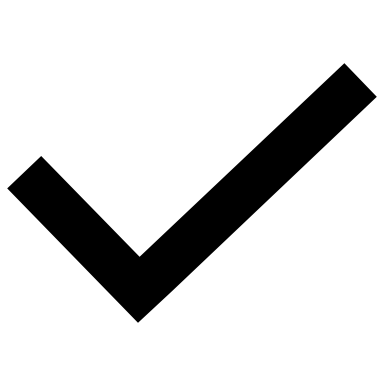 |  | 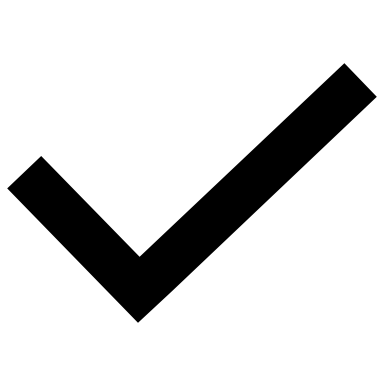 | 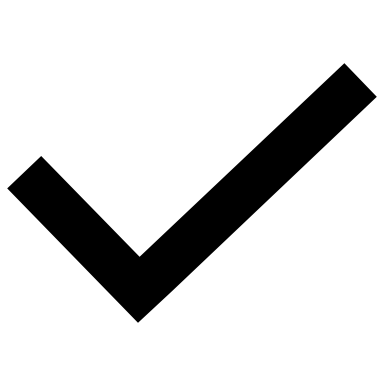 | 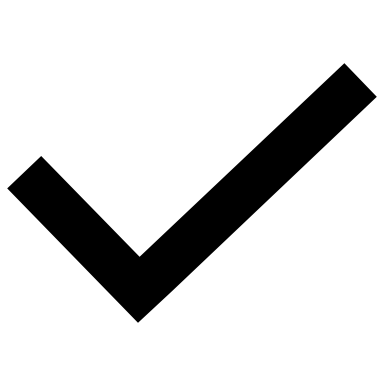 |  |  | 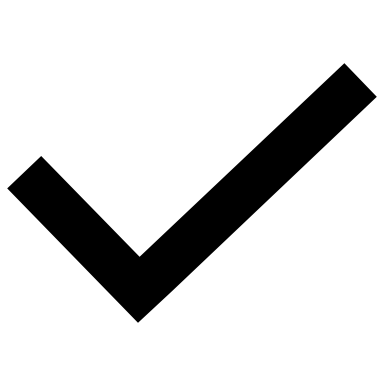 | 13 |
| da Silva et al. (2008) | 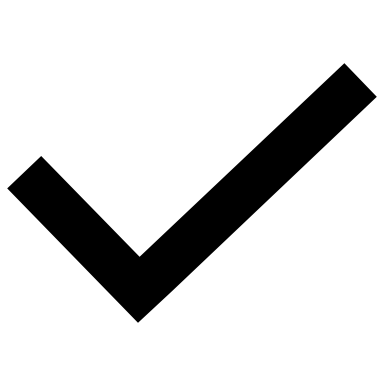 | 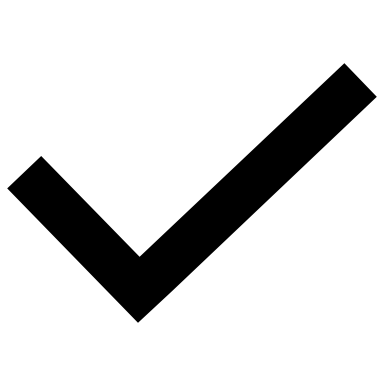 | 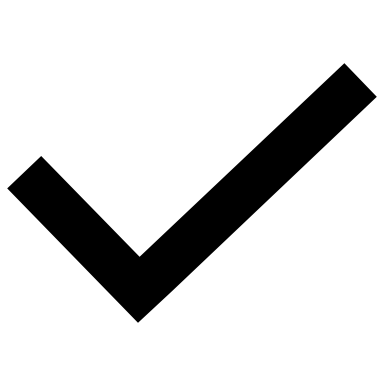 | 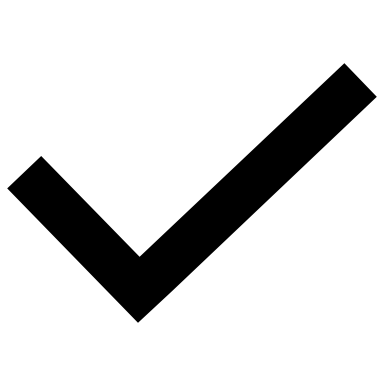 | 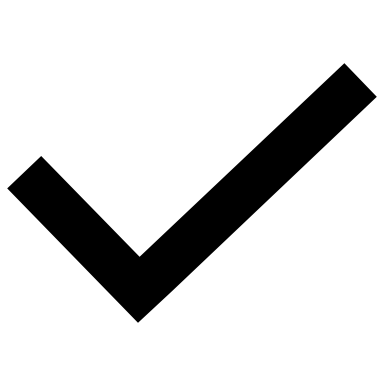 | 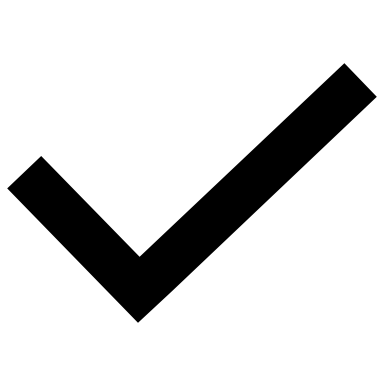 | 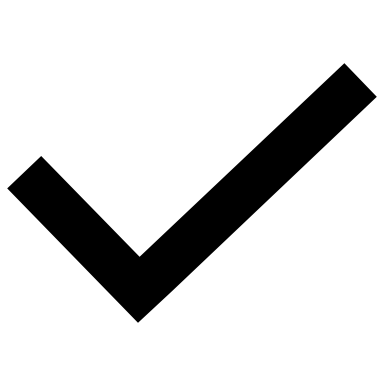 | 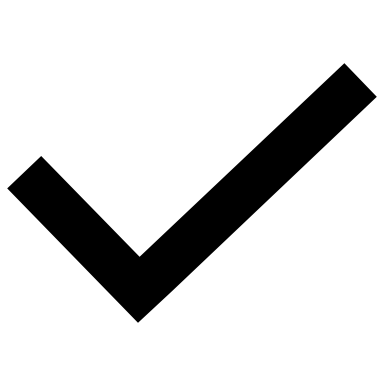 | 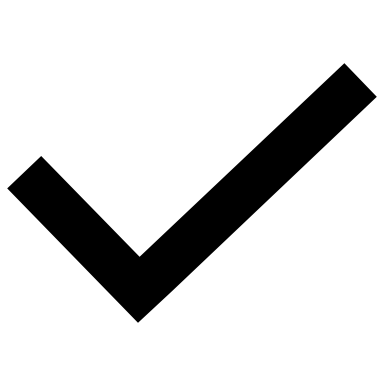 | 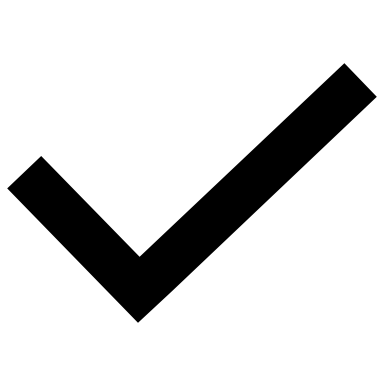 | 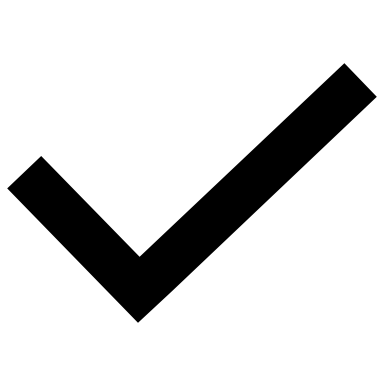 |  | 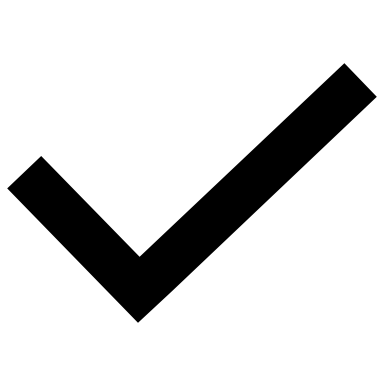 | 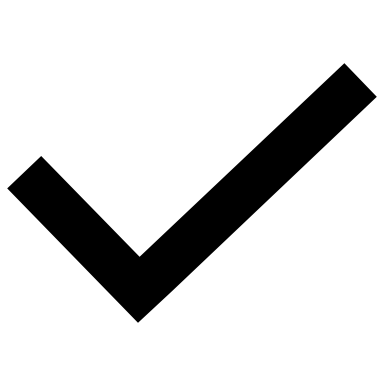 |  | 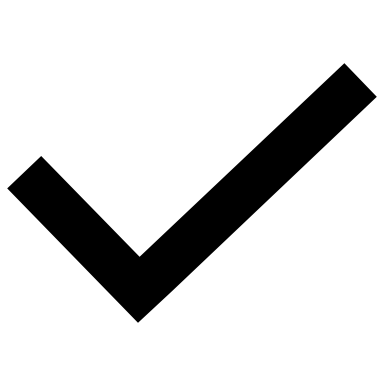 | 14 |
| Rachman et al. (2009) | 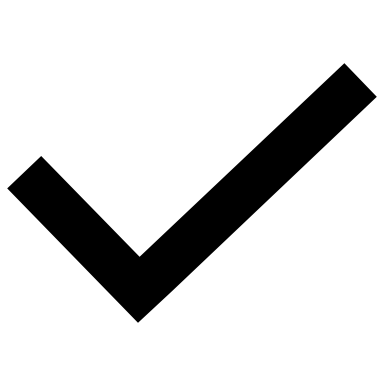 | 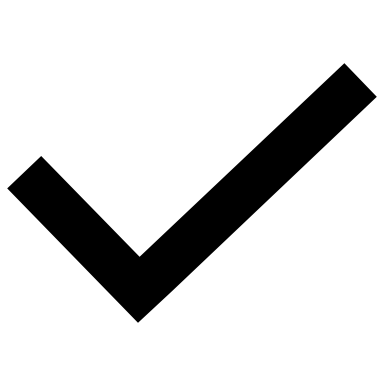 | 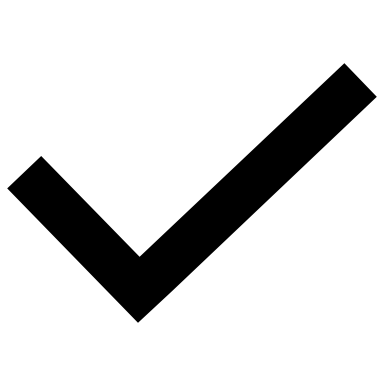 | 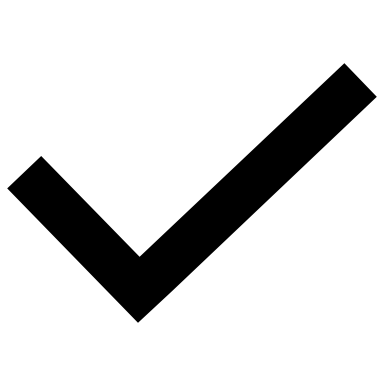 | 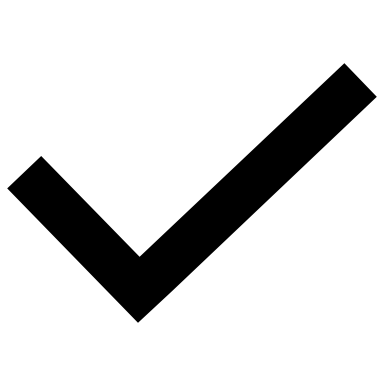 | 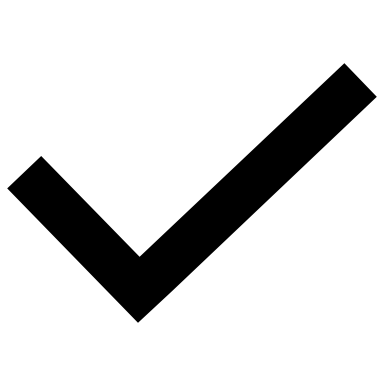 | 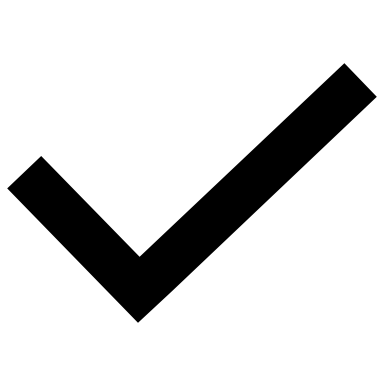 | 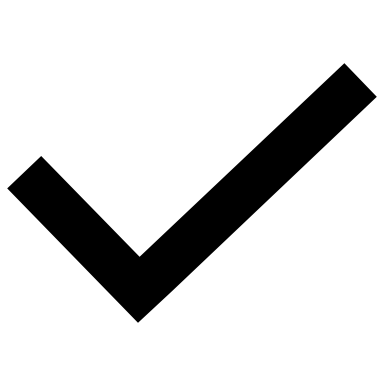 | 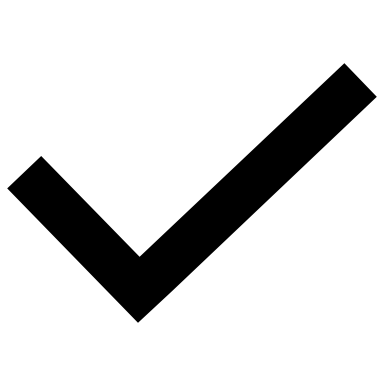 | 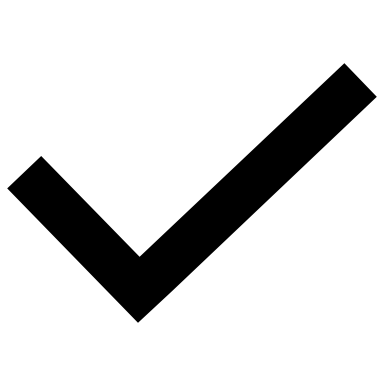 | 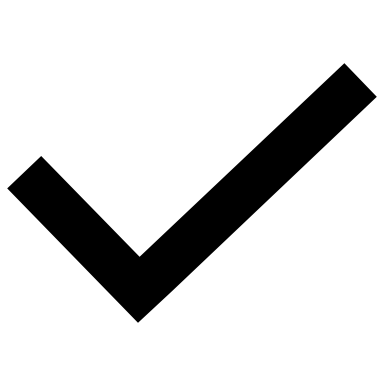 |  | 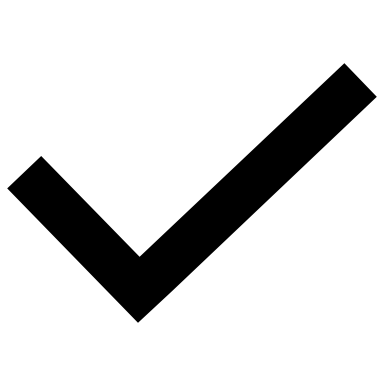 |  |  | 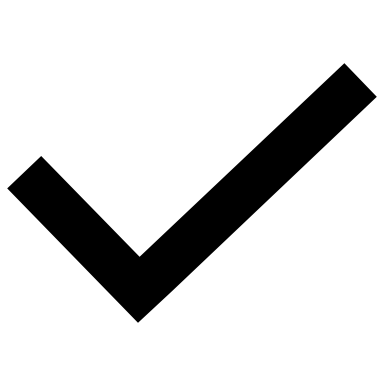 | 13 |
| Kaufman et al. (2012) | 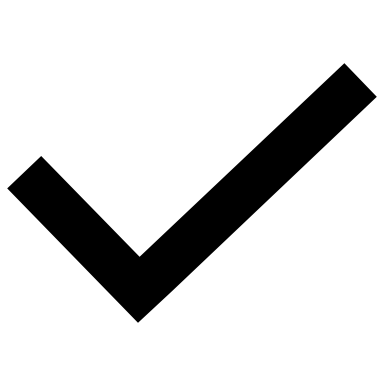 | 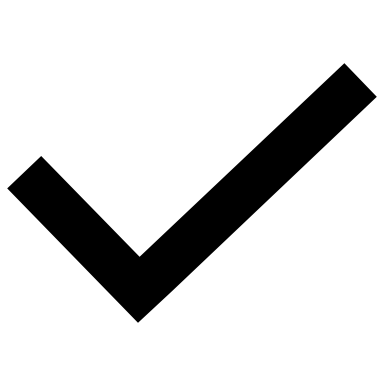 | 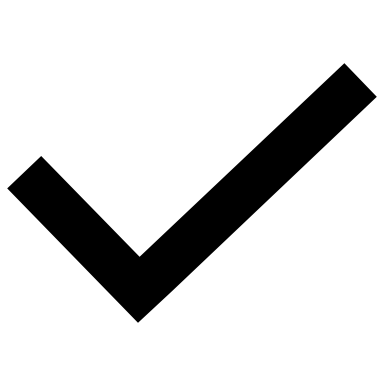 | 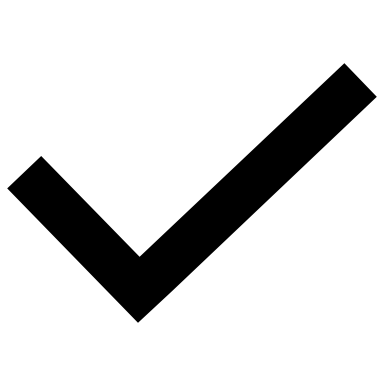 | 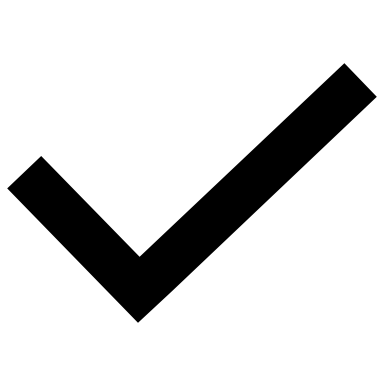 | 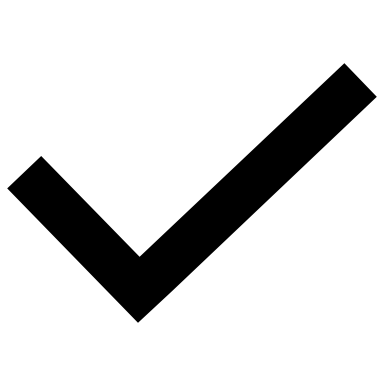 | 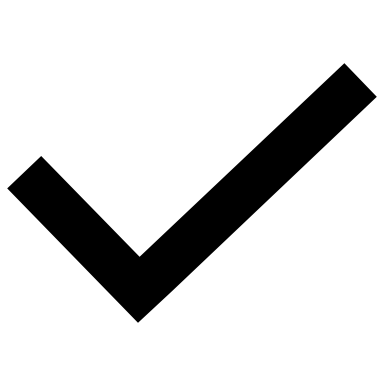 | 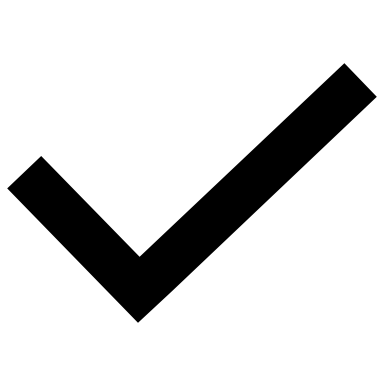 | 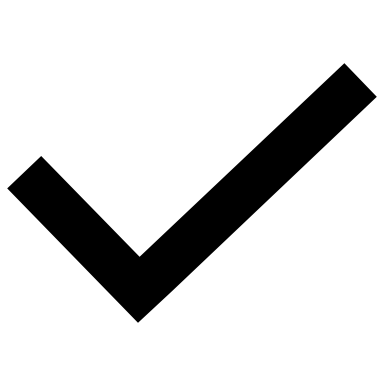 | 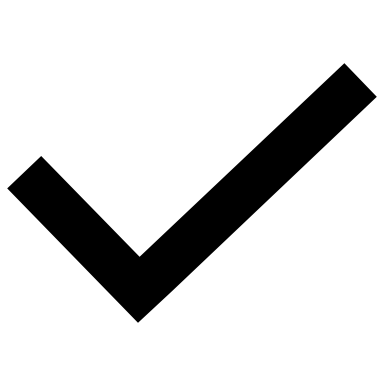 | 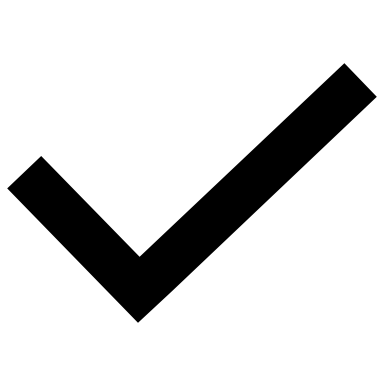 | 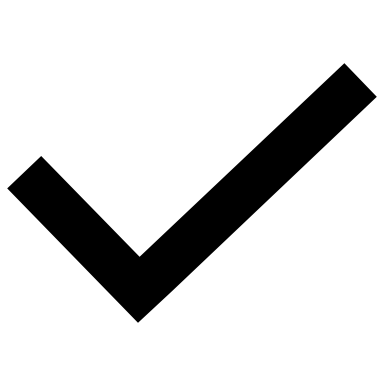 | 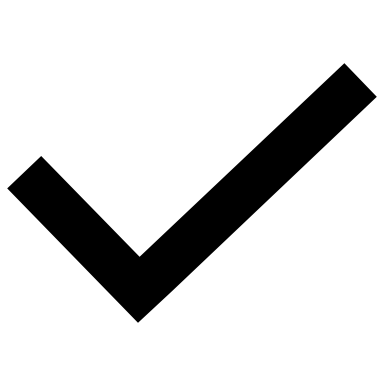 | 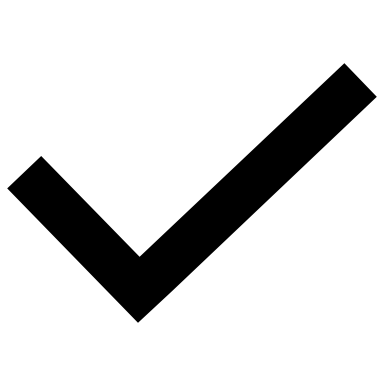 | 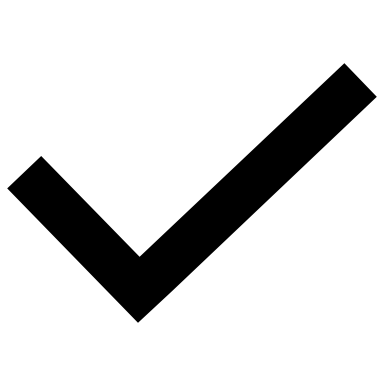 | 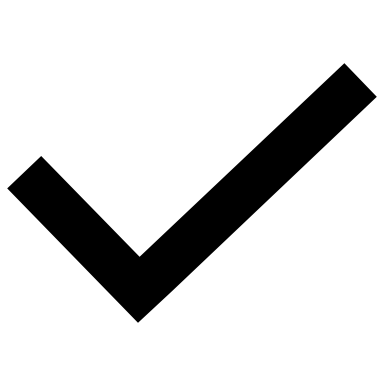 | 16 |
| Meregalli et al. (2013) | 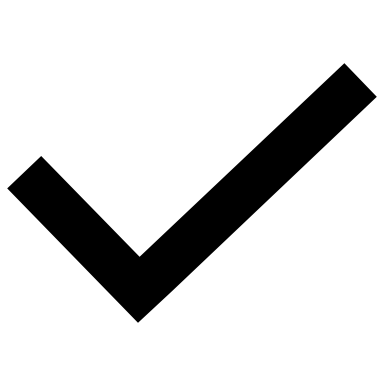 | 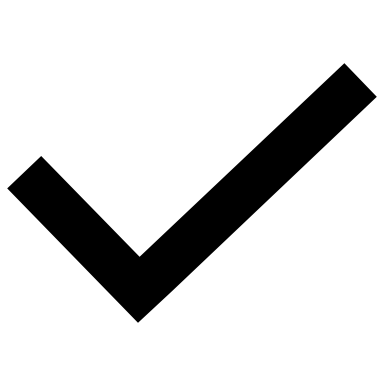 | 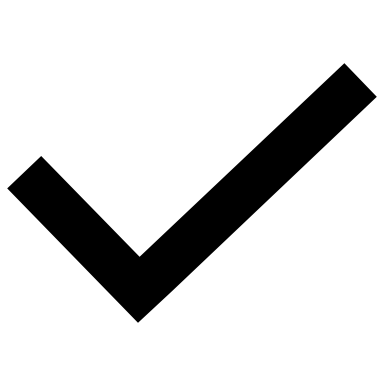 | 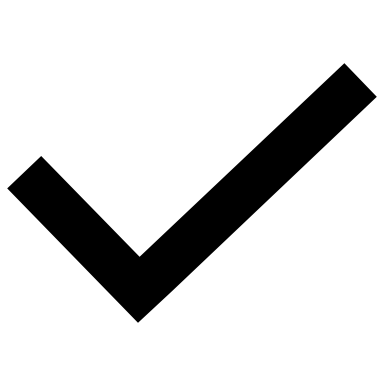 | 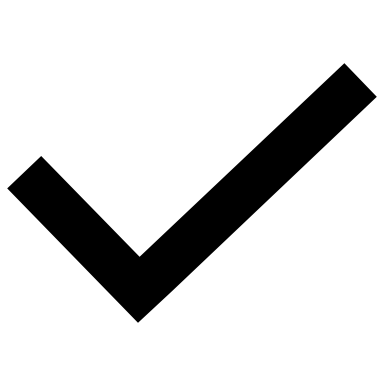 | 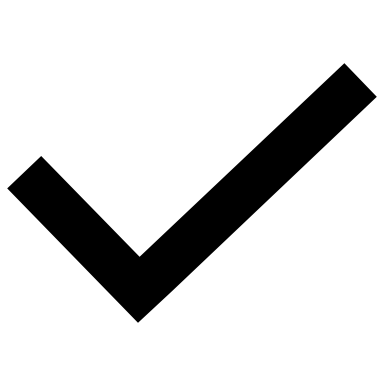 | 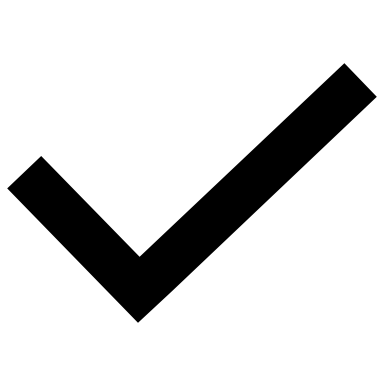 | 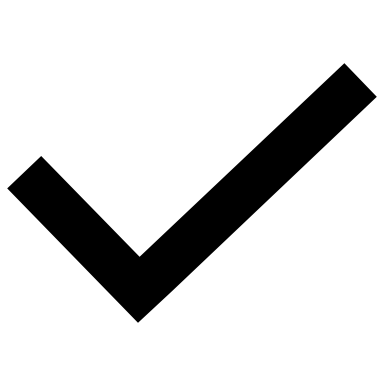 | 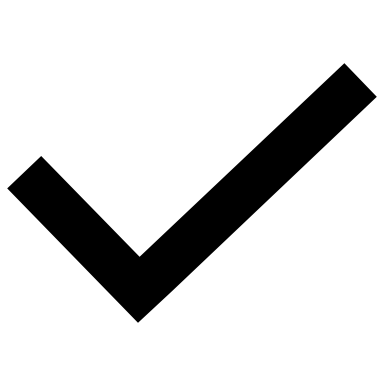 |  | 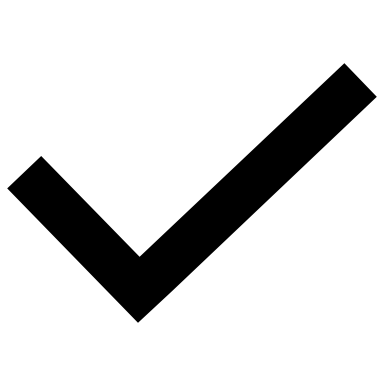 |  |  |  |  | 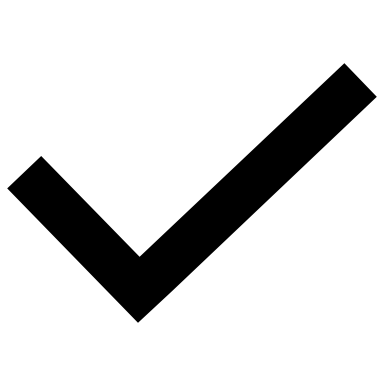 | 11 |
| Menon et al. (2015) | 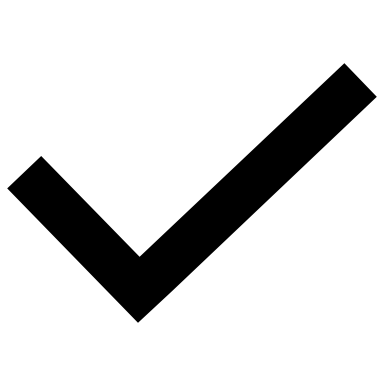 | 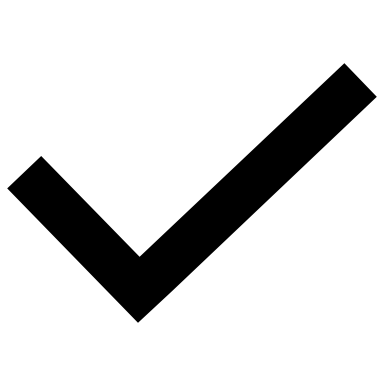 | 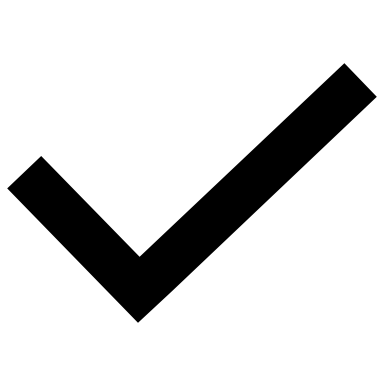 | 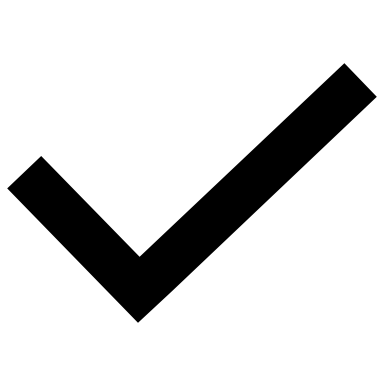 | 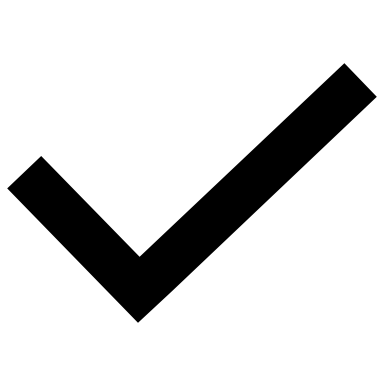 | 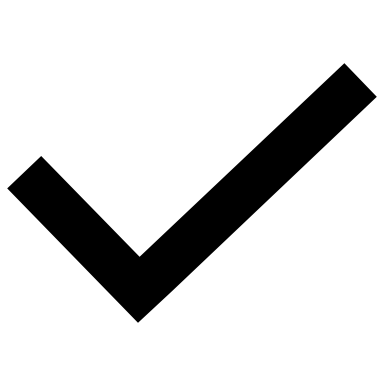 | 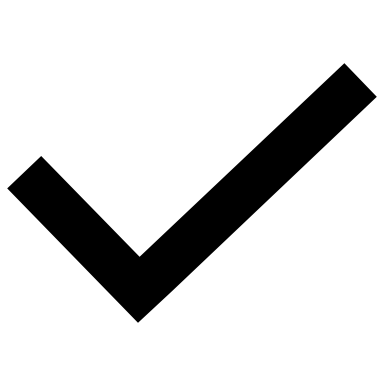 | 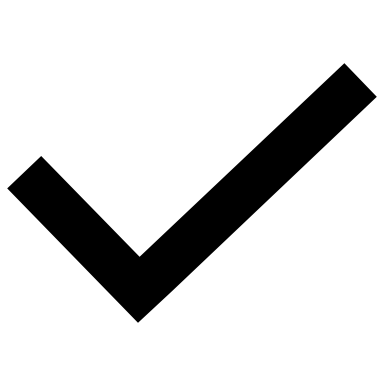 | 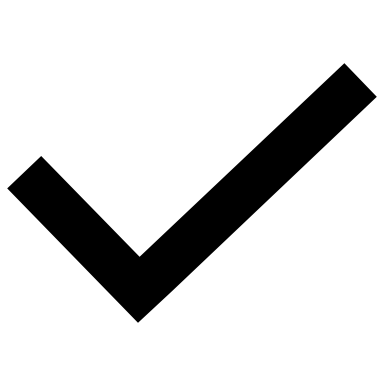 | 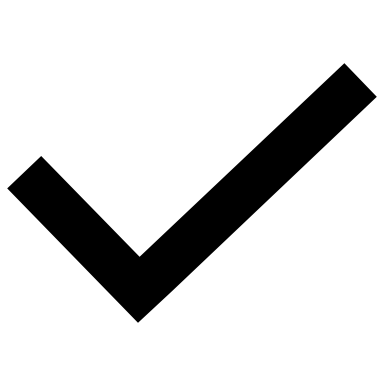 | 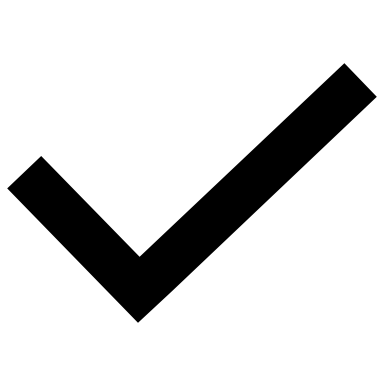 | 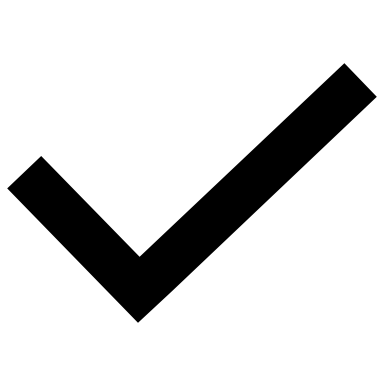 | 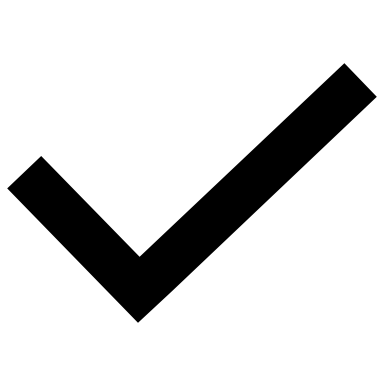 | 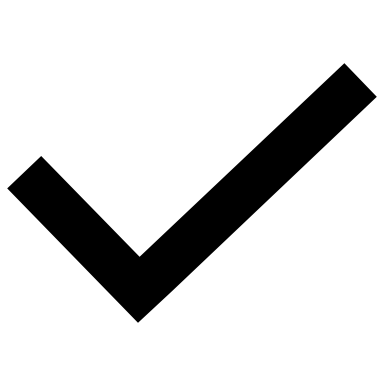 | 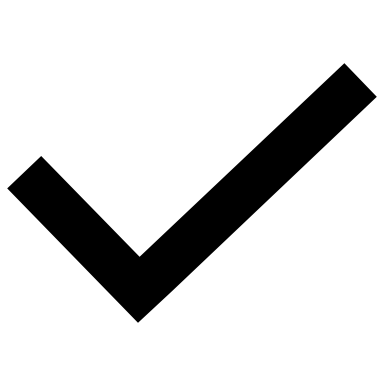 | 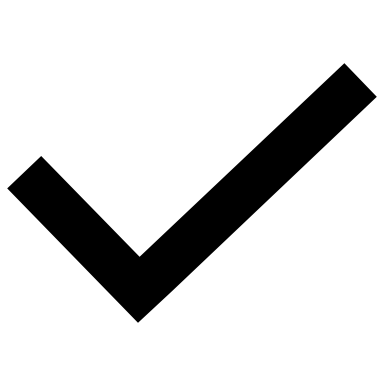 | 16 |
| Tripathi et al. (2015) | 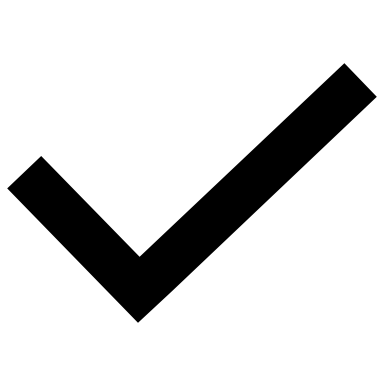 | 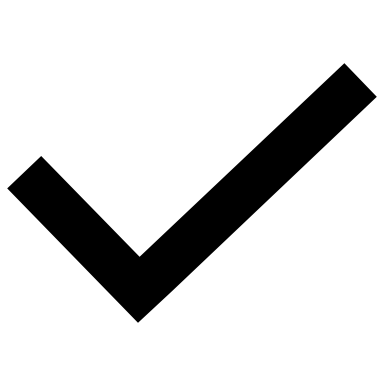 | 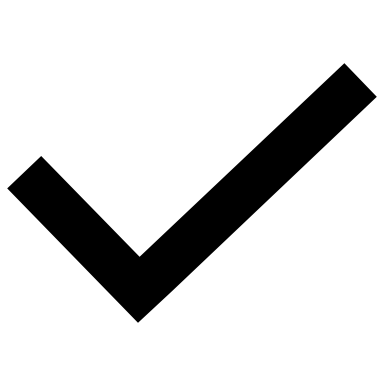 | 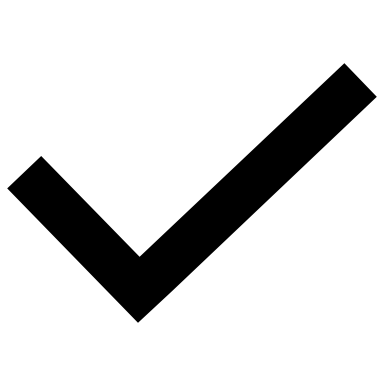 |  |  |  |  |  |  |  |  |  |  |  |  | 12 |
| Al-Abdwani et al. (2018) |  |  |  |  |  |  |  |  |  |  |  |  |  |  |  |  | 10 |
| Kandil et al. (2018) |  |  |  |  |  |  |  |  |  |  |  |  |  |  |  |  | 16 |
| Klugman et al. (2020) |  |  |  |  |  |  |  |  |  |  |  |  |  |  |  |  | 14 |
